# The fNIRS evaluation of frontal and temporal lobe cortical activation in Chinese first-episode medication-naïve and recurrent depression during a verbal fluency task

**DOI:** 10.3389/fpsyt.2023.1132666

**Published:** 2023-04-11

**Authors:** Ting Yang, Hongyu Wang, Haiyue Dai, Juan Hui, Jintong Zhang, Juan Li, Guimei Cui, Juan Wang, Junlin Mu, Zhaohui Zhang

**Affiliations:** ^1^Department of Neurology, The First Affiliated Hospital of Xinxiang Medical University, Xinxiang, China; ^2^The Second Affiliated Hospital of Xinxiang Medical University, Xinxiang, China; ^3^Henan Key Laboratory of Neurorestoratology, The First Affiliated Hospital of Xinxiang Medical University, Xinxiang, China

**Keywords:** functional near-infrared spectroscopy, VFT, major depressive disorder, first-episode, recurrent

## Abstract

**Background:**

Functional near-infrared spectroscopy (fNIRS) identifies neurophysiological differences between psychiatric disorders by assessing cortical hemodynamic function. Few trials have studied differences in brain functional activity between first-episode medication-naïve depression patients (FMD) and recurrent major depression (RMD). We aimed to determine the differences between FMD and RMD in oxygenated hemoglobin concentration ([oxy-Hb]), and to investigate the correlation between frontotemporal cortex activation and clinical symptoms.

**Methods:**

We recruited 40 patients with FMD, 53 with RMD, and 38 healthy controls (HCs) from May 2021 to April 2022. Symptom severity was assessed with the 24-item Hamilton Depression Rating Scale (HAM-D) and the Hamilton Anxiety Rating Scale (HAM-A). A 52-channel fNIRS measured changes in [oxy-Hb] during VFT performance.

**Results:**

Both patient groups performed poorly during the VFT task compared with HC (FDR *p* < 0.05), but there was no significant difference between the two patient groups. Analysis of variance showed that mean [oxy-Hb] activation was lower in both the frontal and temporal lobes in the MDD group compared with HCs (FDR *p* < 0.05). Additionally, patients with RMD had a significantly lower hemodynamic response in the right dorsolateral prefrontal cortex (DLPFC) and dorsal frontal pole cortex (DFPC) than patients with FMD (FDR *p* < 0.05). No significant correlation was found between changes in mean [oxy-Hb] and either medical history or clinical symptoms (FDR *p* < 0.05).

**Conclusion:**

The presence of different neurofunctional activity in some of the same brain regions in FMD and RMD patients implied a link between the level of complexity activation in frontal regions and the stage of MDD. Cognitive impairment may already be present at the beginning of an MDD episode.

**Clinical trial registration:**

www.chictr.org.cn, identifier ChiCTR2100043432.

## 1. Background

Major depression disorder (MDD) is a common global psychiatric disorder marked by low mood, loss of interest or pleasure, anhedonia, and low self-worth (1). The current prevalence is about 2–4% worldwide and 1.7–2% in China (2). Depression is projected to have the highest burden of disease worldwide by 2030 (3). MDD has a serious impact on cognition, and cognitive impairment was shown to affect one or more areas of executive function at the onset of depression, including learning, memory, and attention, which remained in remission (4). MDD is a highly recurrent disease, and 50–60% of patients who experience a first depressive episode experience recurrences (5), and number of relapses is associated with the severity of MDD (6). Cognitive impairment, such as delayed verbal memory, is more severe in patients with recurrent major depression (RMD) and worsens with increase in the number of depressive episodes (7).

Differences between first-episode medication-naive depressed patients (FMD) and RMD have been described. One study found that patients with RMD were more likely to experience anxiety, fatigue, and sleep problems that affected their quality of life and increased the risk of recurrent episodes compared with FMD patients (8). Sheets et al. (9) used factor analysis to identify three personality factors, interpersonal hypersensitivity, antisocial conduct and social anxiety, as predictors of RMD. A recent study found that cognitive impairment occurred during the first depressive episode, and that as the number of episodes increased, patient performance in cognitive domains such as processing speed, working memory, and verbal learning became worse (10). It is clear that the challenge for people with depression is to prevent relapse, but differences between the brain mechanisms of patients with FMD and with RMD are not clear.

Previous studies have shown differences in brain function between FMD and RMD. A functional magnetic resonance imaging (fMRI) study identified different blood oxygenation-level dependent (BOLD) signals in frontoparietal region of patients with FMD and RMD, increased activation of the right inferior frontal gyrus and bilateral middle frontal gyrus and decreased activation of the right middle frontal gyrus in patients with RMD (11). A similar study also found regional homogeneity (ReHo) was higher in the right inferior frontal triangular gyrus and lower in the left inferior temporal gyrus in RMD than in FMD (12). Lu et al. (13) found reduced activation in the right middle frontal gyrus, right thalamus, and right superior temporal gyrus in RMD compared with FMD when the patients performed a sad face recognition task. In addition, multichannel NIRS results showed significantly lower brain activation in the right prefrontal and superior temporal cortex during the verbal fluency task (VFT) in patients with RMD compared with healthy controls and FMD (14). In conclusion, these results suggested significant differences between patients with first-onset depression and recurrent depression in frontal and temporal lobe regions. Owing to the heterogeneity of study methods and subjects, which has complicated the results of previous studies, the differences in brain function between FMD and RMD need further study.

Functional near-infrared spectroscopy (fNIRS) is an emerging functional neuroimaging modality which may be particularly suited as a diagnostic and efficacy prediction tool for psychiatric disorders. The technology can continually monitor hemodynamic changes in the cerebral cortex using near-infrared light (15). Wavelengths of near-infrared light have the unique property of passing through tissues until it reaches the cortex, where it is preferentially absorbed by oxy-hemoglobin and deoxy-hemoglobin (16). Although fNIRS can only measure cortical regions, it is safe, non-invasive, non-restrictive and tolerant to motion. Therefore, it is often used for the direct observation of hemodynamic changes in psychiatric patients during cognitive tasks (17). The combined fMRI-NIRS study demonstrated that the fMRI-measured blood oxygen level-dependent signal and the NIRS measured changes in hemoglobin concentration showed excellent concordance (18). Therefore, fNIRS can be used as a suitable alternative to fMRI for used to study cortical neural activity during engagement in cognitive tasks. The cognitive task commonly used worldwide to trigger prefrontal cortex (PFC) activation in fNIRS studies is the VFT (19, 20). In China, the Chinese version of VFT is used clinically to detect changes in brain function during multichannel fNIRS measurements in patients with psychiatric disorders (21–23). Many studies have shown that the changes in [oxy-Hb] in depressed patients differ from those in healthy controls (HCs) during VFT (24, 25). Patients with MDD have significantly reduced frontal activation when they perform the VFT, which appears to be a reliable biomarker. However, current studies have focused on differences in brain area activation in depressed compared with healthy individuals performing the VFT (26, 27), and less on changes in brain function following multiple episodes of depression. It is thus necessary to study the activation of brain areas in FMD and those with RMD using the Chinese version of VFT.

We hypothesized that hypoactivation of the frontotemporal cortex is typical of patients with MDD, and the degree of frontotemporal [Oxy-Hb] activation differs between FMD and RMD. The primary study aim was to use fNIRS to explore differences of changes in the features of specific cortical regions in HCs, and patients with FMD and RMD when performing the Chinese version of VFT. fNIRS may identify biomarkers that predict the recurrence of depression and add the pathogenesis of what is known of depressive episodes. The secondary aim was to investigate the relationship between cortical [oxy-Hb] and clinical factors, probing the brain areas associated with symptom severity.

## 2. Methods

### 2.1. Participants

Outpatients and inpatients with MDD at the Second Affiliated Hospital of Xinxiang Medical University, Xinxiang, Henan, China, were recruited between May 2021 and April 2022. Experienced psychiatrists diagnosed 40 first-episode medication-naïve MDD and 53 recurrent MDD patients following the criteria of the Structured Clinical Interview for Diagnostic and Statistical Manual of Mental Disorders, fifth edition. For all patients, we used the 24-item Hamilton Depression Rating Scale (HAMD-24) to assess the severity of depression. We used the 14-item Hamilton Anxiety Rating Scale (HAMA-14) to measure anxiety levels. The inclusion criteria were (1) 18–60 years of age, (2) HAMD-24 score ≥ 20, (3) ≥9 years of education, and (4) right-handed. The exclusion criteria were (1) severe and unstable physical illness, (2) received electroconvulsive therapy within 1 year, (3) family history of mental disorders, (4) a history of drug/substance abuse or addiction, (5) pregnancy or breastfeeding, and (6) do not agree to participate in the fNIRS study.

Thirty-eight HC participants were recruited from the local community. They were all right-handed, free of medication, between 18 and 60 years old, and had a senior middle school or higher education. An experienced psychiatrist evaluated each participant to exclude mental illness using the International Neuropsychiatric Interview. Those with long-term substance abuse, severe medical illness, or cognitive impairment were excluded. The Ethics Committee of the Second Affiliated Hospital of Xinxiang Medical University (Xinxiang, Henan, China) approved the study (No. XYEFYLL2021-27), which was registered with the Chinese Clinical Trials Registry (ChiCTR2100043432). Each participant gave informed consent.

### 2.2. VFT

**FIGURE 1:**
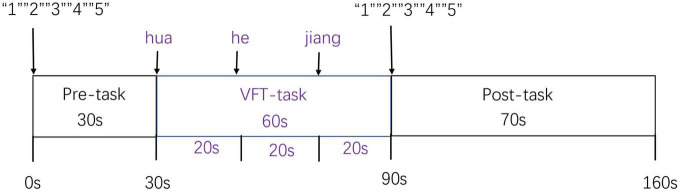
The verbal fluency test (VFT) was divided into the periods, a 30 s pre-task baseline period, a 60 s VFT task period, and a 70 s post-task baseline period.

Before the trial began, participants practiced the VFT with the Chinese character “大,” which means “big.” The exercise was necessary to ensure that participants understood the task and responded correctly to the prompts. The task paradigm included a 30 s pre-task baseline period, a 60 s task period, and a 70 s post-task baseline period. During the pre- and post-task periods, participants were asked to count from one to five repeatedly and aloud. The 60 s task period was divided into three sequential 20 s blocks. During each block, one of three Chinese syllables (“花,” “河,” or “江”) was audibly presented to the participants, who were then asked to generate as many phrases or four-character idioms as possible, speaking softly (Figure 1). The phrases or four-character idioms included a syllable. All participants heard the same syllable cues in the same order. The number of words generated during the task measured task performance. Valid words were recorded by the same researcher.

### 2.3. The fNIRS measurement

**FIGURE 2:**
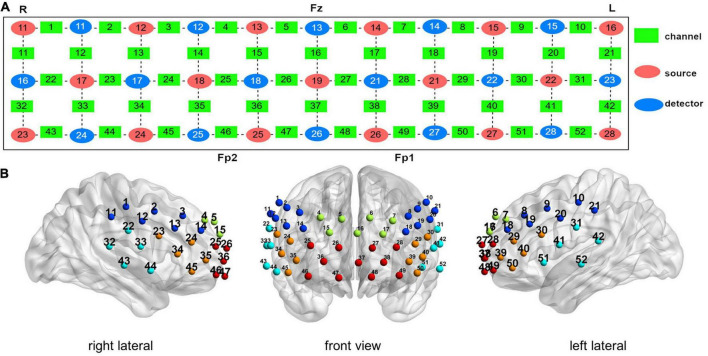
Measurement points of the 52-channel near-infrared spectroscopy (NIRS) system. **(A)** Arrangement of the channels was based on the international 10–20 system. **(B)** Three-dimensional detection region of each channel. (Green: dorsal frontal pole cortex. Red: ventral prefrontal cortex. Orange: ventrolateral prefrontal cortex. Dark blue: dorsolateral prefrontal cortex. Light blue: superior temporal gyrus).

Before recording fNIRS measurements, participants were asked to sit straight on a comfortable chair in a quiet room. To avoid artifacts, participants focused on a red dot on the wall about 2 m in front of them, and were told to minimize blinking and head, body and mouth movements during the VFT. The VFT was performed using a 52-channel NIRS system (ETG4100, Hitachi Medical Corporation, Tokyo, Japan) and two wavelengths of near-infrared light (695 nm and 830 nm) to measure relative changes in [oxy-Hb] based on a modified Beer–Lambert law. The probes, including 17 emissions and 16 detectors, forming a 52-channel configuration, were covered on the bilateral frontal and temporal cortices, and fixed with 3 × 11 thermoplastic shells. The distance between the emission and the detector probes was 3.0 cm, and the area measured between the probes was defined as a channel (Figure 2A). The sampling frequency was 10 Hz. The probe was placed at the center of the forehead in the lowest channel along the Fp1–Fp2 line according to the international 10–20 system (Figure 2B). The corresponding spatial information for each channel in the Montreal Neurological Institute space was evaluated by NIRS_SPM (version 4.0) (28).

### 2.4. The fNIRS signal analysis

Near-infrared spectroscopy data were analyzed by NIRS-SPM.^1^ System noise and physiological changes were removed with the hemodynamic response function (HRF) and a wavelet-minimum description length (MDL)-based detrending method (29). To analyze the NIRS data, changes in [oxy-Hb] were used as the activation data. Changes in [oxy-Hb] more directly reflect cognitive activation than changes in [deoxy-Hb], as shown by a stronger correlation with blood oxygenation level-dependent signals measured by fMRI (30). We performed multiple comparisons using the false discovery rate (FDR) (31) and corrected for neural activation of the 52-channel probes. The significance level was *p* < 0.05. We used the BrainNet Viewer to visualize the brain network (32).

### 2.5. Statistical analysis

Differences in demographic and clinical variables were compared with appropriate tests. Continuous variables were tested by one-way analysis of variance or Student’s *t*-test and categorical variables by chi-square tests. To compare the differences in the level of [oxy-Hb] and VFT scores between groups of participants, a one-way analysis of covariance (ANCOVA) was employed after adjusting for age and duration of illness. One-sample *t*-test were used to compare within-group task-related [oxy-Hb] vs. [oxy-Hb] = 0 (null hypothesis). The combined equivalent dose was calculated for patients who received more than one drug in each class (33). The reference drugs for each class were fluoxetine, diazepam, and chlorpromazine. Spearman’s correlation coefficient (rho) was used to test the relationships between mean changes of [oxy-Hb] and VFT performance, clinical scale scores, duration of illness, and other clinical variables including drug dose in both the FMD and RMD groups. A *p* < 0.05 were considered statistically significant. The statistical analysis was performed with Statistical Product and Service Solutions (SPSS) version 25.0 (IBM Corp., Armonk, NY, USA).

## 3. Results

### 3.1. Participant characteristics

The clinical characteristics of the three groups are shown in Table 1. Differences in sex, age, years of education, HAMD score, HAMA score, and age at onset were not significant. Differences in the duration of illness (*t* = −5.590, *p* < 0.001) and number of episodes (*t* = −8.919, *p* < 0.001) were significant.

**TABLE 1:** Demographic and clinical characteristics of the study participants [mean (S.D.)].

	FMD (*n* = 40)	RMD (*n* = 53)	HC (*n* = 38)	*P*
Age	34.08 (12.80)	31.79 (8.59)	29.34 (5.40)	0.056
Sex (M/F)	11/29	19/34	19/19	0.116
Years of education	13.5 (3.37)	13.55 (3.80)	14.55 (3.13)	0.235
VFT	7.85 (3.83)	8.79 (3.62)	11.89 (4.99)	0.001
Duration of illness (year)	0.91 (0.93)	3.26 (2.86)		<0.001
Age of onset (year)	33.31 (13.17)	28.53 (8.84)		0.052
Number of depressive episodes	1	2.429 (1.46)		<0.001
HAM-D	35.48 (8.36)	35.58 (9.34)		0.953
HAM-A	26.98 (9.77)	24.55 (8.19)		0.196
Fluoxetine eq. dose (mg/day)		23.73 (12.18)		
Diazepam eq. dose (mg/day)		9.38 (7.56)		
Chlorpromazine eq. dose (mg/day)		26.57 (51.96)		

### 3.2. VFT performance

There were significant differences in the number of words generated during the VFT (*F* = 8.196, *p* = 0.001) by the FMD, RMD, and HC groups (Table 1). Notably, VFT performance differed among the three groups when age was included as a covariate (*F* = 10.11, *p* < 0.001). *Post hoc* pairwise comparisons showed that performance was worse in the FMD (*p* = 0.000) and RMD (*p* = 0.002) groups than in the HCs. The difference in the performance of the FMD and RMD groups was not significant (*p* = 0.083).

### 3.3. Hemodynamic response during VFT

**FIGURE 3:**
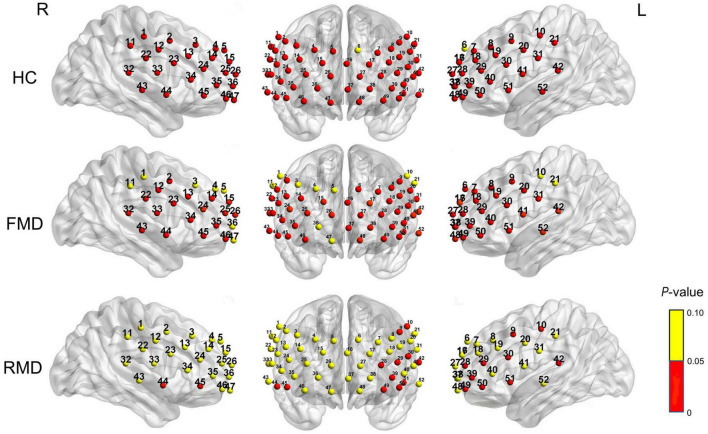
Brain activation based on [Oxy-Hb] during the VFT in the healthy participants **(top row)**, first-episode medication-naïve depressive disorder group **(second row)**, and the recurrent major depressive disorder group **(bottom row)**. Significance (*p* < 0.05) is shown by color shown by the bar on the right. R, right; L, left; FMD, first-episode medication-naïve depression patients; RMD, recurrent major depression; HC, healthy controls.

A significant increase in the [oxy-Hb] changes occurred during the task period in 43 channels,(all except 1, 3, 4, 5, 10, 11, 21, 36, and 47; FDR *p* < 0.05; 23 channels over the left hemisphere and 18 channels over the right hemisphere, excluding medial channel 37, 16) in the FMD group. In the RMD group, there were 12 channels with significant increases of [oxy-Hb] (9, 10, 28–30, 39, 42, 44–45, and 49–51, FDR *p* < 0.05; 10 channels over the left hemisphere and two channels over the right hemisphere). In the HCs, except for channel 6, all the others were significantly active (FDR *p* < 0.05), as shown in Figure 3.

### 3.4. Comparison of NIRS activation

**FIGURE 4:**
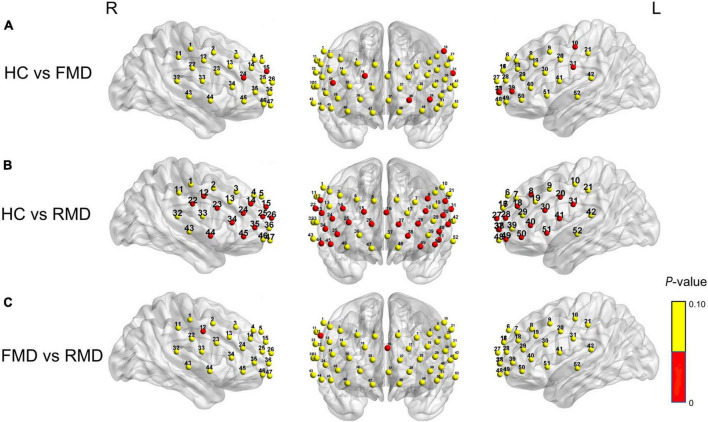
**(A)** FMD group had significantly lower activation of [oxy-Hb] than the HC group in six channels (10, 15, 24, 31, 38, and 39; *p* = 0.001–0.005). **(B)** RMD group had significantly lower activation of [oxy-Hb] than the HC group in 26 channels, (8, 12, 14–16, 18, 20, 22–28, 39, 31, 34, 35, 38, 40, 41, 44, 45, and 49–51; *p* = 0.000–0.007). **(C)** RMD group had significantly lower activation of [oxy-Hb] than the FMD group in two channels (12 and 16; *p* = 0.020–0.025). (Red channels indicate significant differences when comparing between groups). R, right; L, left; FMD, first-episode medication-naïve depression patients; RMD, recurrent major depression; HC, healthy controls.

We found prefrontal activation during VFT was significantly different among the three groups in 27 channels (8, 10, 12, 14–16, 18, 20, 22–28, 30, 31, 34, 35, 38, 40, 41, 44, 45, and 49–51; *F* = 3.181–12.615; *p* = 0.000–0.022; FDR *p* < 0.05; 14 channels over the left hemisphere and 12 channels over the right hemisphere, excluding medial channel 37, 16). The results of *post hoc* tests revealed that the FMD group had significantly lower activation of [oxy-Hb] than the HC group in six channels (10, 15, 24, 31,38, and 39; *p* = 0.001–0.005; FDR *p* < 0.05). Compared with the HC group, patients with RMD had lower [oxy-Hb] activation in 26 channels (8, 12, 14–16, 18, 20, 22–28, 30, 31, 34, 35, 38, 40, 41, 44, 45, and 49–51; *p* = 0.000–0.007; FDR *p* < 0.05). When duration of illness was included as a covariate, the RMD group demonstrated significant hypoactivation relative to FMD at 2 channels during the VFT (12 and 16; *p* = 0.020–0.025; FDR *p* < 0.05), as shown in Figure 4.

### 3.5. Correlation analysis

In patients with FMD, mean [oxy-Hb] changes had a significant positive correlation with the VFT performance in five channels (3, 9, 11, 21, and 50; rho = 0.320–0.367; uncorrected *p* = 0.020–0.045). In patients with RMD, VFT performance was positively correlated with mean [oxy-Hb] changes in six channels (10, 13, 25, 29, 36, and 42; rho = 0.273–0.349; uncorrected *p* = 0.010–0.048). Before the FDR correction, FMD group mean [oxy-Hb] changes and the duration of illness were negatively correlated in one channel (29; rho = −0.373; uncorrected *p* = 0.018), and age at onset were positively correlated in one channel (20; rho = 0.404; uncorrected *p* = 0.010). Age of onset, duration of illness, and number of depressive episodes were not significantly associated with mean [oxy-Hb] changes in the RMD group. However, none of correlations remained significant after FDR correction (FDR *p* < 0.05). In the FMD group, HAMA score and [oxy-Hb] activation were positively correlated in channel 23 (rho = 0.337; uncorrected *p* = 0.333), but not with the HAMD-24 scores. The mean [oxy-Hb] in the RMD group changes were positively correlated with HAMD-24 scores in channel 11 (rho = 0.278; uncorrected *p* = 0.046) and the HAMA scores in channel 46 (rho = 0.298; uncorrected *p* = 0.032). We did not find significant correlations of mean [oxy-Hb] changes with antidepressant, anxiolytic, or antipsychotic drug dose. There were also no significant correlations with any of the 52-channels after FDR correction.

## 4. Discussion

To the best of our knowledge, this is the first fNIRS study to investigate changes in brain cortical activation measured by [oxy-Hb] during VFT performance in Chinese patients with FMD and RMD. The results showed that patients with MDD spoke significantly fewer words in the VFT compared with controls, but here were no significant differences between the two VFT groups. Second, there was significant [oxy-Hb] hypoactivation in various regions of the PFC in both the FMD and RMD groups compared with the HC group. We also compared FMD with RMD patients. Decreased cerebral activation in the right dorsolateral prefrontal cortex (DLPFC) and dorsal frontal pole cortex (DFPC) occurred in the RMD but not in the FMD group.

Patients with MDD spoke significantly fewer words during the VFT compared with controls, which is consistent with evidence that patients with MDD have cognitive impairment (14, 34). Previous studies reported that cognitive dysfunction developed with recurrent episodes of depression (35), but we did not find differences in word formation between FMD and RMD patients during the VFT. That is consistent with previous NIRS results and similar tasks (11, 36). The nullification effect may be explained by the presence of cognitive impairment before the first depressive episode or adverse outcome of depression. Another possibility is that in most NIRS studies, the short time settings of the VFT did not allow for detecting group differences in task performance (26). Collectively, our results showed that patients with MDD had more pronounced cognitive impairment than the HCs, and that differences in cognitive impairment in FMD and RMD were not significant.

We observed significant increases of the mean [oxy-Hb] in 51 channels in HCs, in the bilateral frontotemporal lobes, which consistent with most previous fNIRS studies (37–40). Those studies found PFC activation in healthy individuals during VFT, showing that the HC group was a suitable control for the patient group. In the FMD group, most channels were activated. Hypoactivation was seen only in the RDLPFC. In the RMD group, only 12 channels were activated during the VFT, and most were in the left side of the hemisphere. Low activation occurred in the DFPC, ventrolateral prefrontal cortex (VLPFC) and DLPFC on the right side all showed extra. The study results led us to conclude that the reduced brain activation in the frontal-temporal lobe during VFT in patients with MDD compared with the HCs indicated decreased frontal–temporal lobe function in depressed patients. The result is consistent with previous studies (41, 42) may be a neuropathological basis for cognitive dysfunction in depressed patients. We also found that activation was lower in the right side of the brain in the MDD group, which was in line with prior studies (13, 43). Wang et al. (14) speculated that only the left hemisphere was activated in patients with recurrent depression, which implies that there was compensatory activity in the right hemisphere to overcome the deficit in the left hemisphere during the first-episode of MDD. Unfortunately, with a long disease duration, the level of activation in patients with RMD was significantly reduced, and only the left hemisphere was activated because antidepressants interfered with compensatory mechanisms. The opposite effect was reported in some studies, with a relative reduction in left prefrontal [oxy-Hb] changes during VFT in patients with MDD (44). A whole-head near-infrared spectroscopy study found that resting-state functional connectivity was lower between the left dorsolateral prefrontal cortex and the parietal lobe, but higher between the right orbitofrontal cortex and the lateral prefrontal cortex in patients with MDD (45). Thus, hypoactivation of the right brain region during the VFT showed that asymmetries in hemispheric activation were common in patients with MDD.

We had a very encouraging discovery that patients with RMD had significantly lower mean [oxy-Hb] changes in channel 12 (the right precentral gyrus, PreCG) and channel 16 (the middle frontal gyrus, orbital part, ORBmid) during the VFT compared with patients with FMD, and the channels located in RDLPFC and DFPC. The DLPFC is involved in executive function, including executive control of working memory, thinking activity, and executive control of behavior (46, 47). It is also involved in cognitive control and voluntary or effortful regulation in emotional states (48). The orbitofrontal cortex is associated with emotion regulation, decision making and sensory integration in patients with MDD (49). Previous studies found abnormalities in the DLPFC (50) and DFPC (51) brain regions in patients with MDD. In a neuroimaging study, BOLD signals in the FMD and RMD differed in DLPFC (11), which was consistent with our findings that both MDD and RMD patients showed hypoactivation in the RDLPFC, although patients with RMD had more severe damage in the RDLPFC. Wang et al. (14) found that patients with RMD had significantly lower oxyhemoglobin activation in the dorsolateral prefrontal cortex and pars triangularis Broca’s area than FMD patients, which was partially consistent with our findings. A study that contradicted our results found that the ALFF in treatment-refractory depression with persistence and relapse was significantly higher in the orbitofrontal cortex than it was in HCs (52). Despite the difference in findings, they help to clarify the neuropathological mechanisms of MDD, and the lower activation in the DLPFC and DFPC brain regions in patients with RMD may be due to the long-term recurrent nature of the disease.

The VFT was not associated with a significant correlation between mean [oxy-Hb] and depression severity in either the FMD or the RMD group. That was consistent with previous studies. Huang et al. (53) did not find a significant correlation between HAMD scores and [oxy-Hb] concentration. Because of a small sample size, Tsujii et al. could not find a significant correlation between HAMD scores and [oxy-Hb] concentration (54). Many studies have reported both negative (26, 55) and positive (56) correlations. Again, unfortunately, we also did not find a correlation between channel activation and anxiety symptoms in either group. Hu et al. (43) found that the HAMA scores was negatively correlated with [oxy-Hb] changes in two channels in the left ventrolateral prefrontal cortex in all participants. One study (56) found a significant positive correlation between the severity of anxiety symptoms and changes in [oxy-Hb]. In this study, the correlation between [oxy-Hb] activation and the severity of clinical symptoms was uncertain. Future studies may require recruitment of more participants at different clinical stages to clarify this uncertainty.

As the RMD patients were on medications, we evaluated correlation of the use of various classes of medications with changes in [oxy-Hb], but none were significant. In a cross-sectional study, stepwise regression to control for any confounding factors influencing frontal centroid value did not find significant associations of clinical variables with the frontal centroid value (25). Large studies of psychiatric patients treated with various medications did not find strong effects on fNIRS signals (57). However, a randomized controlled trial (58) studied the impact of sedative antidepressants on fNIRS signals. They reported that mirtazapine (15 mg for 8 days) added [oxy-Hb] values to the VFT period, but trazodone (25 mg for 8 days) and the placebo had no significant effect on the fNIRS signals. Reduced activation of the left prefrontal cortex and the right premotor cortex during verbal tasks in patients with MDD treated with paroxetine was found in the fMRI study (59). However, Takamiya et al. (60) found differences in the depression scores in the high-dose and low-dose groups after treatment were not significant. In group comparisons, NIRS signals were significant for high-dose compared with low-dose antidepressants. Currently, the mechanisms behind the clinical efficacy of these treatments remain elusive. Differences between the results of various studies may have resulted from variations in patient characteristics and in the methods used to study the changes in [oxy-Hb] revealed by fNIRS. More thorough experimental designs and methods are required to confirm these mechanisms in the future.

## 5. Conclusion

The VFT revealed cognitive impairment in all patients with FMD and RMD. The study also confirmed the presence of asymmetry in brain activation in patients with MDD and significant differences in the signals of RDLPFC and DFPC brain regions under fNIRS measurements in FMD and patients with RMD. Our results confirmed the relationship between the [oxy-Hb] signal in the aforementioned brain regions and MDD. It also showed that MDD-specific pathology needed further study. The activation patterns measured by fNIRS were used to aid clinical diagnosis. The findings increase our understanding of the pathogenesis and clinical predictors of patients with MDD. RDLPFC and DFPC are expected to be important targets for interventions to prevent recurrent episodes of MDD.

## 6. Limitations

One of the study limitations was that all patients with RMD were taking medications and we could not exclude the influence of medication and anxiety disorders on the psychiatric disorders. However, for ethical and safety reasons, it was considered unacceptable to recruit patients with RMD and not allow them to receive medication. Regardless, we did not find any relationships between mean [oxy-Hb] changes and drug dose in the RMD group. Also, we included the first-episode in patients without medication, which might have an original pattern of cognitive impairment in patients with FMD. Secondly, unlike fMRI and PET, there is no standard analysis of NIRS data, and different research groups have performed analyses based on different complex analysis tools (61, 62). Additionally, the number of episodes and duration of illness was inconsistent in the RMD group, and studies of first episodes in patients with RMD appear have greater research value. Finally, our sample size was small, some differences between the two MDD groups may have been significant if the sample size had been larger. Despite these limitations, the findings provide evidence of a better diagnosis and prognosis for patients with FMD and RMD in the future.

## Data availability statement

The raw data supporting the conclusions of this article will be made available by the authors, without undue reservation.

## Ethics statement

The studies involving human participants were reviewed and approved by the Second Affiliated Hospital of Xinxiang Medical University (Xinxiang, Henan, China). The patients/participants provided their written informed consent to participate in this study. Written informed consent was obtained from the individual(s) for the publication of any potentially identifiable images or data included in this article.

## Author contributions

TY, JL, and ZZ designed the study. TY, HW, HD, and GC collected the data. TY, JH, and HW analyzed the experiment data. TY drafted the manuscript. TY, JM, JZ, JW, and ZZ approved the final version of the manuscript. All authors contributed to the article and approved the submitted version.

## Footnotes

1
https://www.nitrc.org/projects/nirs_spm/
